# Open-mindedness trait affects the development of intercultural communication competence in short-term overseas study programs: a mixed-method exploration

**DOI:** 10.1186/s12909-022-03281-2

**Published:** 2022-03-30

**Authors:** Chen Wang, Shuang-Ying Wu, Yi-Zi Nie, Guan-Yu Cui, Xiang-Yu Hou

**Affiliations:** 1 Center for Brain, Mind and Education, Shaoxing University, Shaoxing, 312000 China; 2 School of Teacher Education, Shaoxing University, Shaoxing, 312000 China; 3 School of Psychology and Counselling, Faculty of Health, Queensland University of Technology, Brisbane, 4059 Australia; 4 Department of Psychology, School of Education, Wenzhou University, Wenzhou, 325035 China; 5 Poche Centre for Indigenous Health, The University of Queensland, Brisbane, 4067 Australia

**Keywords:** Intercultural communication competence, Open-mindedness, Healthcare students, Overseas study programs, Mixed-method

## Abstract

**Background:**

Overseas study trips can enhance healthcare students’ intercultural communication competence. An opportunity to immerse in the new culture enables them to develop their ability to offer services to people from different countries. However, the role that open-mindedness (i.e., a personality trait) can play in this process has not been explored.

**Methods:**

The present study adopted a mixed-method design to identify how open-mindedness trait affected this overseas learning process. Thirty-two undergraduate healthcare students in Australia took part in the study. Questionnaires, which measured socio-demographic information, intercultural communication competence and open-mindedness trait were administered to the participants before and after their overseas trip. Half of the participants (*n* = 16) were interviewed after the overseas trip.

**Results:**

The correlational analysis showed that the open-mindedness trait was correlated with cultural skills, a component of intercultural communication competence, but not significant with the other three components. Three themes emerging from the qualitative data indicated that the open-mindedness trait affected students’ cultural exposure. This trait enabled participants to be actively involved in the immersion in the local culture. They were willing to learn from peer fellows, and keen to embrace novel challenges.

**Conclusion:**

It is concluded that open-mindedness trait is vital for increasing cultural immersion, and hence promote intercultural communication skills.

## Background

Intercultural communication competence (ICC) is an essential capability for health professionals, which has been defined as the ability to communicate effectively and appropriately in intercultural situations [1]. In healthcare settings, the majority of patient adverse events have been caused by inappropriate communication [2]. However, interventions to improve communication skills should be considered in a social context with a potential danger of miscommunication [3], such as in a multicultural society and intercultural situations. It has great significance to research on the development of ICC among healthcare professionals/students who need to encounter intercultural situations.

Research in the field of education usually uses the short term “intercultural competence” to refer to intercultural communication competence [1], and there are several synonym terms in other fields. For example, research in the field of health utilises the term “cultural competence/competency” [4, 5], whereas research within the field of business usually uses the term “cultural intelligence (CQ)” [6]. Although each of these fields has developed its own definition and key terms, the underlying theories are quite similar. Another synonymous is cross-cultural competence. According to a classical comparison made by Lusting and Koester, “intercultural communication involves interactions among people from different cultures, whereas cross-cultural communication involves a comparison of interactions among people from the same culture to those from another culture” [7]. Based on this comparison, ICC is more suitable in the present study.

In the research of ICC, the KASA model has been widely used [8]. Based on this model, ICC consists of four components, knowledge (K), attitude (A), skills (S), and awareness (A +) [9]. Intercultural communication skills of health professionals can affect the quality of healthcare delivery and outcomes [10, 11]. To assist students’ future careers, universities provide cultural education for healthcare students to develop their ICC [12–15]. Short-term overseas study programs, a typical type of cultural education, have been regarded to be the most effective way to increase students’ cultural exposure and hence develop their ICC [16–19]. It has great practical significance to research on how to make full use of the overseas programs.

In addition to cultural education, personality, especially open-mindedness trait, has been suggested to be a vital factor to affect ICC. Personality traits determine an individual’s behaviour patterns by affecting one’s intrapersonal processes, such as emotional, motivational, and cognitional processes [20]. Although several traits are supposed to be highly related to intercultural success [21], open-mindedness has been agreed to be the most important trait to contribute to ICC [21, 22]. Open-mindedness trait refers to an individual’s open attitude to cultural values and norms different from their own, and an unprejudiced attitude to the group members in the different cultures [21]. Theoretically, researchers have discussed the foundation for the association between personality traits and ICC, suggesting that open-mindedness (a typical social-related trait) helps an individual to respond to threatening intercultural situations with positive affect [23, 24]. Empirically, extensive research has revealed a positive correlation between open-mindedness and ICC [25–28].

Given the stability of the personality of university students, this area lacks sufficient research on the open-mindedness trait from the perspective of the educational process. Personality traits are relatively stable in a short period, and can hardly be changed immediately after an education intervention [29]. Usually, previous research focused on the effects of specific cultural learning programs [13, 30, 31], such as short-term overseas study programs, and open-mindedness trait is simply considered as a control variable [32]. It is unknown whether and how the open-mindedness trait can affect the development of ICC from overseas study experiences. However, according to Kolb’s theory of experiential learning, learners “must be able to involve themselves fully, openly, and without bias in new experiences” in order to achieve effective learning from experiences [33, 34]. According to the definition of open-mindedness trait, open-minded learners are just those who are able to involve themselves openly and without bias in new experiences [21]. Therefore, it could be assumed that open-minded learners tend to have more effective experiential learning, leading to more learning outcomes. In the overseas programs, students are actually “learning by doing”, which is typically a kind of experiential learning [17, 30]. Therefore, it is hypothesized that open-mindedness is associated with the changing tendency of ICC, that is, the learning outcome of short-term overseas study programs. Taking Kolb’s theory of experiential learning as a theoretical foundation, the present study aimed to test this hypothesis using a mixed-method research design.

## Methods

### Research design

A mixed-method approach was adopted in the present study, collecting both quantitative and qualitative data. The quantitative data were collected via a pre- and post-test survey. The qualitative data were collected via individual interviews. Ethical approval was obtained from the University Human Research Ethics Committee of the University of [BLINDED] (Approval Number: 1500000662, 1,600,000,806) before the study was conducted. Consent was implied by the return of a completed survey and written informed consent was obtained for the interviews.

### Research setting and sample

The present study was conducted in the Faculty of Health at the University of [BLINDED] in Australia. The Faculty provides short-term overseas study programs for their students to gain cultural learning and professional growth in a different country. The study sample included a total of 32 undergraduate healthcare students who were recruited from several such programs. Because such programs were usually held during the summer/winter holidays and there was only a limited number of healthcare students participating in the programs each year, the data collection lasted about two years (2015–2017). Students in the sample were not first-generation immigrates or international students. In these programs, students went to a south Asian country to undertake a health placement for two to four weeks in the local universities, hospitals, or other health organisations.

English is not the official language in these south Asian countries, but some of the health professionals in the host countries can speak English. Although there were formal or informal translators in the working places, language barriers existed in the communication with the majority of the local people. Therefore, the students in the present study had to encounter both linguistic and cultural differences.

### Quantitative survey and procedure

A survey was conducted with 32 healthcare students before their departure from (pre-test) and after their return to Australia (post-test). In the pre-test, students completed a questionnaire measuring socio-demographic information, open-mindedness trait, and ICC. In the post-test, only ICC. A self-developed socio-demographic form was used to ask age, gender, school year, religion, ethnicity, family income sufficiency, family language, and whether they spoke a language other than English. Open-mindedness trait was measured by the Open-minded Subscale in the Multicultural Personality Questionnaire – short form (MPQ-SF) [35]. This is a 5-point Likert subscale with eight items (1 = totally not applicable, 5 = completely applicable). Intercultural communication competence was measured by the Cultural Intelligence Scale (CQS) with four subscales [36]. The CQS is a 7-point rating scale (1 = strongly disagree, 7 = strongly agree), and involves 4 items for cultural awareness (i.e., metacognition), 6 items for cultural knowledge (i.e., cognition), 5 items for cultural attitude (i.e., motivation), and 5 items for cultural skills (i.e., behaviour).

### Qualitative interviews and procedure

The 32 students, who completed the quantitative survey, were invited to take part in the individual interviews after the post-test. Eventually, 16 of them accepted the invitation and signed informed consent forms before the interviews. The interviews were semi-structured and audio-recorded, conducted by the leading author. In the interviews, several open-ended questions were designed and asked, during which students were allowed to speak about any aspects of their overseas study experiences and changes of ICC. For example, “How was your overseas experience? (Did anything surprise you about yourself, about the environment, about your profession?) Tell me what you learnt from it.” A professional transcription service was employed to accomplish the transcription verbatim. The leading author then reviewed and edited the transcripts to ensure the accuracy of the data.

### Statistical analysis

SPSS 22.0 was used for the qualitative data analysis. Descriptive statistics were adopted in analysing the socio-demographic information of the sample, and the variables measured by scales. Correlation analysis was conducted to explore the associations between the open-mindedness trait and changed ICC, which was calculated as the post-test scores minus the pre-test scores.

Qualitative data were analysed using thematic analysis. Specifically, the analysis was based on Braun and Clarke’s six key steps of Thematic Analysis: (1) becoming familiar with the transcripts; (2) coding each transcript; (3) searching for general themes based on the initial codes; (4) reviewing and refining themes; (5) defining themes with names; and (6) writing a result report [37].

## Results

### Quantitative data

Table 1 displays the socio-demographic information of the survey sample. All the students were in the second, third, or fourth school year. The majority of them were female, which was consistent with the gender proportion in the Faculty of Health, where more female students undertook bachelor degrees associated with healthcare, especially nursing, public health, and social work, than male students. More than half of them were Caucasian, non-religious, monolingual, and from families speaking English.

**Table 1:** Social-demographic characteristics of the survey sample

	**n**	**Percentage**
**Age**		
18–20	12	37.5%
21–25	8	25.0%
26–30	11	34.4%
31–50	1	3.1%
**Gender**		
Female	28	87.5%
Male	4	12.5%
**University year level**		
1st	0	0.0%
2nd	20	62.5%
3rd	9	28.1%
4th	3	9.4%
**Religion**		
Christian	9	28.1%
Muslim	1	3.1%
Hindu	1	3.1%
Buddhist	0	0.0%
Other	2	6.3%
None	18	56.3%
**Ethnicity**		
Ethnic majority (Caucasian)	19	59.4%
Ethnic minority	13	40.6%
**Speak another language except for English**		
No	20	62.5%
Yes	12	37.5%
**Language spoken in family**		
English	25	78.1%
Others	7	21.9%
**Income sufficiency**		
Very insufficient	2	6.3%
Insufficient	2	6.3%
Just Sufficient	7	21.9%
Sufficient	16	50.0%
Very sufficient	5	15.6%

Before conducting further analysis, the reliability of the scales was tested. The Cronbach Alphas of the CQS are 0.68–0.84 in the pre-test, and 0.89–0.95 in the post-test. The Cronbach Alpha of the MPQ-SF is 0.79. All of the subscales of the CQS and the Open-minded Subscale of the MPQ-SF indicated adequate internal consistency in the present study.

**Fig. 1:**
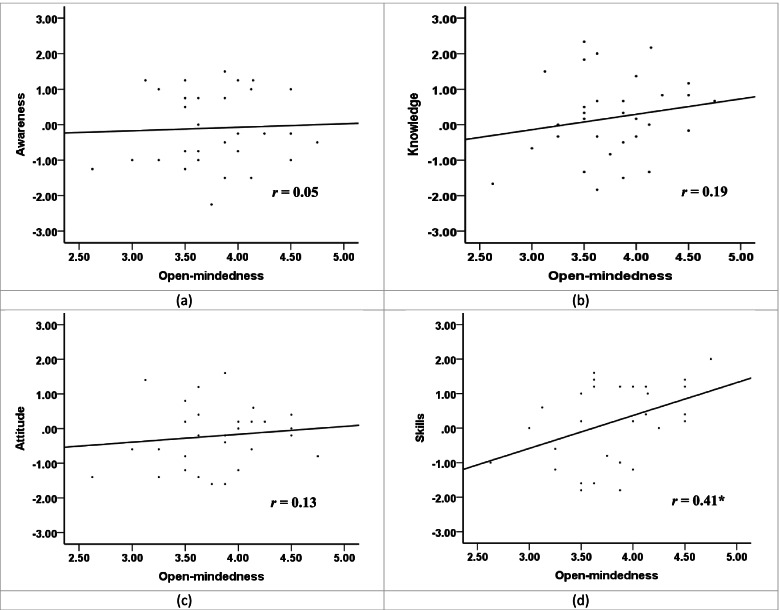
Scatter plots of the correlations between open-mindedness and changed ICC. Note. *r* = correlation coefficients between the changed scores and open-mindedness; * *p* < 0.05

Descriptive statistics of the scale variables are shown in Table 2. Correlation analysis was conducted between open-mindedness and changed ICC, which equals the post-test scores minus the pre-test scores. As shown in Fig. 1, results reveal that the open-mindedness scores are significantly associated with the scores of cultural skills (*r* = 0.41, *p* = 0.019), but not the other components of ICC (*p* > 0.05). That means students with a high level of open-mindedness tend to gain more cultural skills in the short-term overseas study programs.

**Table 2:** Descriptive statistics (*M* ± *SD*)

	**Metacognition-cultural awareness**	**Cognition-cultural knowledge**	**Motivation-cultural attitude**	**Behaviour-cultural skills**	**Open-mindedness trait**
Pre-test	5.28 ± 0.72	3.83 ± 0.97	5.63 ± 0.66	5.12 ± 1.02	3.79 ± 0.48
Post-test	5.19 ± 1.14	4.04 ± 1.05	5.42 ± 1.10	5.29 ± 1.20	-
△	-0.09 ± 1.03	0.19 ± 1.17	-0.21 ± 0.85	0.17 ± 1.12	-

*Note.* △ = post-test scores – pre-test scores (changed scores)

To further identify the specific increased skills related to open-mindedness, the five items of the behavioural subscale in the CQS were analysed (see Table 3). According to the self-rating scores of these items, students with a high level of open-mindedness demonstrated a tendency to develop more on the skills to “change verbal behaviour (e.g., accent, tone)”, and “alter facial expressions” in intercultural communication.

**Table 3:** Correlation analysis between open-mindedness and specific intercultural communication skills

Item	Changed score (M ± SD)	r	p
1. I change my verbal behaviour (e.g., accent, tone) when a cross-cultural interaction requires it	0.38 ± 1.48	0.49^**^	0.005
2. I alter my facial expressions when a cross-cultural interaction requires it	0.19 ± 1.60	0.40^*^	0.025
3. I vary the rate of my speaking when a cross-cultural situation requires it	0.00 ± 1.44	0.31	0.081
4. I use pause and silence differently to suit different cross-cultural situations	0.63 ± 1.46	0.21	0.240
5. I change my non-verbal behaviour when a cross-cultural situation requires it	0.22 ± 1.41	0.14	0.445

Note. *p < 0.05, **p < 0 0.01

### Qualitative data

The thematic analysis of the qualitative data aimed to further explore the possible explanations of why open-mindedness was associated with more development of ICC in short-term overseas study programs. The development of ICC is the outcome of cultural learning. Therefore, more increase in ICC means more cultural learning from the overseas experiences. Three themes about how open-mindedness affected students’ overseas experiences were extracted. The themes suggested that the open-minded students were likely to make full use of the experiences to gain cultural exposure by being *active to immerse in the local culture*, *willing to learn from peers*, and *keen to embrace novel challenges*.

#### Active to immerse in the local culture

Several students who were open-minded were highly active to immerse in the local culture. The open attitude to cultural immersion can bring more communication experiences, which accounts for the development of ICC. They were open to experiencing as much as possible in the host country. Extremely, two students reported that they lived in a homestay, whereas most of their peer fellows in the same program lived in hotels. In their program, the host did not provide accommodation, these two students decided to book a homestay together in order to learn more about the local life. It is a typical example that when encountering the same situation, a student with an open mind has a more active response in action. Their choice in homestays allowed a more continuous cultural immersion, even after they finished activities in the daytime, thus making the most use of the overseas time.

“I got to see the actual lifestyle, I think, a lot more. So we were staying with a woman who had a lot of nephews, so they'd come over on the weekend and we'd play games with them. Yeah, it was really fun. I think I thought I had an idea of the culture before I left…” (P6)

One participant (P2) who had a lot of overseas travelling and study experiences also pointed out directly that the attitude made a difference by saying “if you are always staying in hotels and you just do organised tours, you're not dealing with the locals, and so you are just in this little bubble”.

Because of the language barriers, intercultural communication might start painfully, but it went on better along with the growth of the students’ communication skills over time.

“It just took a lot more time to communicate with them [the lady running the homestay and her family], and I found it exhausting in the first week that I was there taking an hour to say something that usually takes 10 minutes. A little bit painful to keep on trying and rewording things and explaining and if you thought that they'd misinterpreted, having to go back to it again...But it got better as time went on…”(P6)

#### Willing to learn from peers

In the interviews, some students talked about their observations on the peer fellows and demonstrated a willingness to learn from them. The open attitude to observational learning can expand the effect of cultural immersion and then contribute to the development of ICC. Interestingly, the learning occurred more when observing the inappropriate than the appropriate behaviours of their peers in intercultural communications. After observing the peers’ inappropriate behaviours, students had a conscious thought to behave differently from their peers. This consciousness comes out from the peer influence that students try to avoid making the same mistakes.

“Some of the other students were kind of acting like they were still at X [the home university], like sitting on their phones and their computers, and not paying attention... I was really mindful of that the whole time I was there, and I think it was the first time that I really saw how we might be seen by other people. And I was like, "Oh, my God. We look so rude, and we look just so disrespectful” (P9)

In addition to avoiding the same mistakes of their peers, there is another kind of influence to correct mistakes. If a student was told what behaviours were inappropriate and what a better approach could be, this student might change the behaviours. In this way, both the one who noticed the mistake and the one who made the mistake could improve their ICC through peer influence.

“We’re out as a group and we invited some of the Chinese girls [from the host university] to the karaoke… and the other girls even when the Chinese girls were with us, didn't slow down their speaking. So, they were still talking to each other and talking really fast, and then there is this 3rd person and it's like you need to talk slower to include that person which they didn't realise…” (P2).

“I saw a lot of times her [a peer fellow] say something, and they would just look. You could tell they were confused and didn't understand and it just wasn't going in. But she'd keep talking and then I'd have to say, ‘Oh, they didn't get that. Just repeat it or explain it in a different way’.” (P13).

#### Keen to embrace novel challenges

Several students highlighted the open tendency to embrace novel challenges. The open attitude to novel challenges can lead to more cultural immersion and then contribute to the development of ICC. In other words, these students had the inner motivation to step out of their comfort zone, that is, whether they liked to stay in a big group with culturally familiar members. If students stayed within culturally familiar groups, they were still protected in a comfort zone where they did not need to address cultural challenges. For instance, “we were part of a group, so it's not like I had to pick things up by myself and work things out. If I wasn't sure, we were part of a group and we all kind of worked it out together” (P4). Some participants realised the negative effects of cultural shelter when reflecting the overseas experiences.

“I think if you’re going in a group, that can affect it [learning from overseas experiences] as well because you're constantly speaking English with other people around you… you're kinda sheltered cos there is always someone who can speak on your behalf” (P2).

“I was going overseas with all of these people [peer fellows in the program] that have been at university for so long…they never wanted to go up to somebody and ask for directions, or ask to call me a cab, or talk to the cab driver, or stuff like that. So I was always doing that...” (P9)

## Discussion

Based on the quantitative and qualitative data, the present study answered whether and how the open-mindedness trait is associated with the changing tendency of ICC during short-term overseas study programs. The quantitative survey revealed that the open-mindedness trait was associated with increased cultural skills (i.e., the behavioural component of ICC). Although little research has investigated directly the relationship examined in the present study, our results support Kolb’s theory of experiential learning that learners who involve themselves openly in new experiences can achieve more effective learning from experiences [33, 34]. Additionally, our finding of the significant association between open-mindedness and ICC skills is consistent with the evidence found in the related area of cultural adjustment [38, 39]. For example, the research on ethnic minority students suggested that open-mindedness correlates with interethnic interactions and social adjustment [39].

In literature, two possible explanations for the association could be found theoretically. Firstly, an individual with an open mind has been found to hold a positive attitude toward novel experiences [40], which leads to a tendency of having more exposure to a new culture and subsequently gaining more increases in intercultural communication competence. Secondly, reflection has been found to be a key process of learning from experience [41], and students with an open mind probably often reflected on their experiences, whereas the other students did not. However, empirical evidence from the qualitative data in the present study is more likely to support the first explanation. According to previous literature, exposure to other cultures does not always lead to greater intercultural understandings and capabilities [42]. By comparing the previous studies and the present study, two different kinds of exposure have been found. In the passive exposure, students usually position themselves in a social-exclusion space, whereas our results highlighted the role of open-mindedness in promoting ICC via increasing active exposure. It could be further concluded that only active exposure to other cultures is effective to the development of ICC, which is the contribution to the literature by the present study.

To be noted, the association with open-mindedness is displayed on the behavioural component of ICC (i.e., cultural skills), but not on the other components (i.e., cultural awareness, knowledge, and attitude). It could be assumed that cultural awareness, knowledge, and attitude are enhanced by the general exposure in the overseas trips, and not so sensitive to an individual’s open-mindedness as to cultural skills. According to the models of ICC [9, 36, 43], cultural awareness is the consciousness of mental processes that an individual adopts to learn and understand cultural knowledge; cultural knowledge refers to the knowledge of rules and conventions in different cultures, and understanding of similarities and differences across cultures; cultural attitude is the dynamics to learn about and function in a different cultural context; cultural skills reflect the ability to take suitable verbal and nonverbal actions in the cross-cultural interaction. As discussed earlier, being open-minded can increase substantially cultural exposure, and hence an individual has much more opportunities to practice intercultural communication skills directly.

ICC is like a long journey with multiple steps to the final stage when an individual is fully cross-culturally competent [44]. It is not enough for healthcare students to participate in a cultural intervention and expect changes in ICC dramatically, and some of the previous research has even found a decreased tendency of self-rated competence in short-term overseas study programs [32, 45]. From this perspective, it is reasonable that open-mindedness simply contributes to the development of intercultural communication skills in a short period. This issue has not been discussed in previous literature and further research is needed to make more investigations.

### Practical implications

For the less open-minded students, more preparation and knowledge before departure is needed for the short-term overseas study programs. Being thoroughly prepared would assist healthcare students to learn more from these programs. Specifically, students could prepare themselves to be open-minded by understanding cultural differences and respecting those differences. Coordinators of the programs could also inform the students of the findings in the present study, so as to encourage students to have the consciousness of being active to immerse in the local culture, willing to learn from peer fellows, and keen to embrace novel challenges. Furthermore, students could be encouraged to reflect openly on their experiential learning before, during, and after their overseas study.

Additionally, educations could consider some long-term teaching strategies to positively affect students’ open-mindedness traits, such as providing language courses and cultural courses. For example, nursing students in Australia must undertake some courses about cultural safety to obtain their degrees. In such cultural courses, healthcare students open their minds to the world, and hence it is reasonable to become more open-minded in the process.

### Limitations

The present study sheds light on whether and how the open-mindedness trait affects students’ ICC changes from overseas study experiences. However, the study has several limitations. Firstly, the sample was small and limited to Australian students. Future research could consider a large and diverse sample to examine the findings. Secondly, the data were all self-reported. The evaluation of ICC is more like the self-efficacy to be culturally competent, rather than a true capability. Thirdly, the quantitative data were based on a correlation design with little control over other related variables. Future research can consider a more rigorous design and explore the effects of other personality traits. Finally, the present study was conducted before the COVID-19 pandemic. Considering the stress and other related mental issues of healthcare [46], results might not be suitable in this special situation. Overall, it should take caution to generalize and interpret the findings in the present study. 

## Conclusions

Open-mindedness trait can affect the change of ICC during short-term overseas study programs. Specifically, students who are more open-minded tend to gain more increase in cultural skills from their overseas study experiences. A possible explanation is that the open-mindedness trait leads to the high quality of culture-related experiences. Open-minded students are likely to be active to immerse in the local culture, willing to learn from their peers’ behaviours, and keen to embrace novel challenges.

## Data Availability

The datasets generated and/or analysed during the current study are not publicly available due to privacy and ethical concerns, but are available from the corresponding author on reasonable request.

## Abbreviation

ICC: Intercultural communication competence

## References

[1] Deardorff DK (2009). The SAGE handbook of intercultural competence.

[2] Leonard M, Graham S, Bonacum D (2004). The human factor: the critical importance of effective teamwork and communication in providing safe care. Qual Saf Health Care.

[3] Watson B, Mullany L (2020). Communication accommodation theory as an intervention tool to improve interprofessional practice in healthcare. Professional Communication: Consultancy, Advocacy, Activism.

[4] Amerson R (2010). The impact of service-learning on cultural competence. Nurs Educ Pers.

[5] Gozu A, Beach MC, Price EG, Gary TL, Robinson K, Palacio A, Smarth C, Jenckes M, Feuerstein C, Bass EB (2007). Self-administered instruments to measure cultural competence of health professionals: a systematic review. Teach Learn Med.

[6] Ang S, van Dyne L, Koh C, Ng KY, Templer KJ, Tay C, Chandrasekar NA (2007). Cultural intelligence: Its measurement and effects on cultural judgment and decision making, cultural adaptation and task performance. Manage Organ Rev.

[7] Lustig MW, Koester J (1993). Intercultural competence: interpersonal communication across cultures.

[8] Yarosh M, Lukic D, Santibanez-Gruber R (2018). Intercultural competence for students in international jointmaster programmes. Int J Intercult Rel.

[9] Fantini A, Tirmizi A (2007). Exploring and assessing intercultural competence (CSD Research Paper No. 07–01).

[10] Betancourt JR, Green AR, Carrillo JE, Ananeh-Firempong O (2003). Defining cultural competence: a practical framework for addressing racial/ethnic disparities in health and health care. Public Health Rep.

[11] Flynn PM, Betancourt H, Emerson ND, Nunez EI, Nance CM (2019). Health professional cultural competence reduces the psychological and behavioral impact of negative healthcare encounters. Cultur Divers Ethnic Minor Psychol.

[12] Australian Department of Foreign Affairs and Trade. 2021 New Colombo Plan. Available at https://www.dfat.gov.au/people-to-people/new-colombo-plan/about. Accessed 10 Dec 2021.

[13] Chae D, Kim J, Kim S, Lee J, Park S (2020). Effectiveness of cultural competence educational interventions on health professionals and patient outcomes: a systematic review. Jpn J Nurs Sci.

[14] Greenwood S, Fyers K (2018). Epistemological development in first-year nursing students undertaking cultural safety education. J Nur Educ.

[15] Keane E, Provident I (2017). Combining online education with international service learning to increase cultural competence. Internet J Allied Health Sci Pract..

[16] Beach MC, Price EG, Gary TL, Robinson KA, Gozu A, Palacio A, Smarth C, Jenckes MW, Feuerstein C, Bass EB (2005). Cultural competence - A systematic review of health care provider educational interventions. Med Care.

[17] Amerson R (2012). The influence of international service-learning on transcultural self-efficacy in baccalaureate nursing graduates and their subsequent practice. Int J Teach Learn Higher Educ.

[18] Choi JS, Kim JS (2018). Effects of cultural education and cultural experiences on the cultural competence among undergraduate nursing students. Nurse Educ Pract.

[19] Varela OE (2017). Learning outcomes of study-abroad programs: A meta-analysis. Acad Manag Learn Edu.

[20] Burger JM (2011). Personality.

[21] van der Zee KI, van Oudenhoven JP (2001). The multicultural personality questionnaire: reliability and validity of self-and other ratings of multicultural effectiveness. J Res Pers.

[22] Caligiuri P, Tarique I (2012). Dynamic cross-cultural competencies and global leadership effectiveness. J World Bus.

[23] van der Zee K, van Oudenhoven JP (2013). Culture shock or challenge? The role of personality as a determinant of intercultural competence. J Cross Cult Psychol.

[24] Wang C, Shakespeare-Finch J, Dunne MP, Hou X-Y, Khawaja NG (2021). How much can our universities do in the development of cultural intelligence? A cross-sectional study among health care students. Nurse Educ Today.

[25] Ang S, van Dyne L, Koh C (2006). Personality correlates of the four-factor model of cultural intelligence. Group Organ Manage.

[26] Fischer R (2011). Cross-cultural training effects on cultural essentialism beliefs and cultural intelligence. Int J Intercult Rel.

[27] Wang D, Freeman S, Zhu CJ (2013). Personality traits and cross-cultural competence of Chinese expatriate managers: a socio-analytic and institutional perspective. Int J Hum Resour Man.

[28] Wang L, Wang KT, Heppner PP, Chuang CC (2016). Cross-national cultural competency among Taiwanese international students. J Divers High Educ.

[29] Leong CH (2007). Predictive validity of the multicultural personality questionnaire: a longitudinal study on the socio-psychological adaptation of Asian undergraduates who took part in a study-abroad program. Int J Intercult Rel.

[30] Gower S, Duggan R, Dantas J, Boldy D (2019). One year on: Cultural competence of Australian nursing students following international service-learning. J Nur Educ.

[31] Philips L, Bloom T, Gainey T, Chiocca E (2017). Influence of short-term study abroad experiences on community health baccalaureate students. J Nur Educ.

[32] Wang C, Hou X-Y, Khawaja NG, Dunne MP, Shakespeare-Finch J (2021). Improvement in the cognitive aspects of cultural competence after short-term overseas study programs. Int J Environ Res Public Health.

[33] Kolb DA (1984). Experiential learning: Experience as the source of learning and development.

[34] Morris TH (2020). Experiential learning – a systematic review and revision of Kolb’s model. Interact Learn Envir.

[35] van der Zee K, van Oudenhoven JP, Ponterotto JG, Fietzer AW (2013). Multicultural personality questionnaire: development of a short form. J Pers Assess.

[36] Ang S, van Dyne L, Koh C, Ng KY, Templer KJ, Tay C, Chandrasekar NA (2007). Cultural intelligence: Its measurement and effects on cultural judgment and decision making, cultural adaptation and task performance. Manage Organ Rev.

[37] Braun V, Clarke V (2006). Using thematic analysis in psychology. Qual Res Psychol.

[38] Yakunina ES, Weigold IK, Weigold A, Hercegovac S, Elsayed N (2012). The multicultural personality: does it predict international students' openness to diversity and adjustment?. Int J Intercult Rel.

[39] Corradi D, Levrau F (2021). Social adjustment and dynamics of segregation in higher education - Scrutinising the role of open-mindedness and empathy. Int J Intercult Rel.

[40] McCrae RR, Costa PT, Pervin LA, John OP (1999). A five-factor theory of personality. Handbook of personality: Theory and research.

[41] Carrington S, Selva G (2010). Critical social theory and transformative learning: evidence in pre-service teachers' service learning reflection logs. High Educ Res Dev.

[42] Tsui ABM, Scarino A, Murray N (2014). English as lingua Franca on campus: cultural integration or segregation?. Dynamic Ecologies: A Relational Perspective on Languages Education in the Asia-Pacific Region.

[43] Campinha-Bacote J (2002). The process of cultural competence in the delivery of healthcare services: a model of care. J Transcult Nurs.

[44] Campinha-Bacote J (2007). The process of cultural competence in the delivery of healthcare services: the journey continues.

[45] Kohlbry PW (2016). The impact of international service-learning on nursing students' cultural competency. J Nurs Scholarsh.

[46] Pasam T, Pasam C, Dake R, Soren DK (2021). Incidence of depression, anxiety and sleep disorders in healthcare personal after the onset of Covid 19 pandemic - a survey based study. Crit Care Innov.

